# Reviewed article: Fonseca MEO, Azevedo FKSF, Silva ALR, Pepato MA, Brito WI, Pavan E, Fontes CJF, Oliveira RG. Healthcare-associated infections in an intensive care unit: a descriptive study, Mato Grosso state, Brazil, 2018-2021. Epidemiol Serv Saude. 2026:35;e20250405

**DOI:** 10.1590/S2237-96222026v35e20250405.a

**Published:** 2026-03-30

**Authors:** Mariana Del Grossi Moura

**Affiliations:** 1 Instituto Sírio-Libanês de Ensino e Pesquisa, São Paulo, SP, Brazil

## First round comments

Agradecemos a submissão do manuscrito RESS-2025-0405 intitulado “Análise do perfil de resistência microbiana e estágios em unidade de terapia intensiva: estudo retrospectivo em Mato Grosso, Brasil” à Revista Epidemiologia e Serviços de Saúde: revista do SUS (RESS).

Após a revisão do seu artigo, identificamos a necessidade de uma Revisão maior (Major Revision) para que o manuscrito esteja de acordo com os padrões editoriais da revista e para viabilizar sua publicação.

Além dos pontos detalhados pelos revisores, reforço:

1. Título: Deve informar o tema principal, o delineamento, o local e o(s) ano(s) da pesquisa, em consonância com o guia de redação aplicável da revista.

2. Resumo estruturado: Precisa ser revisto quanto ao conteúdo, à forma e à ordem das informações. Deve conter até 250 palavras e seguir a estrutura: Objetivo, Métodos, Resultados, Conclusão.

3. Palavras-chave: Inserir cinco termos a partir da lista Descritores em Ciências da Saúde (DeCS), disponível em: http://DeCS.bvs.br. Apresentar a primeira letra de cada palavra em maiúscula, conforme os DeCS. O último descritor deve se referir ao delineamento do estudo.

4. É necessário garantir consistência entre os objetivos, métodos, resultados e conclusões, tanto no resumo quanto no corpo do texto.

5. Recomenda-se a adequação rigorosa ao checklist STROBE, destinado ao relato de estudos observacionais, disponível em: https://www.strobe-statement.org/.

6. Discussão: O primeiro parágrafo deve interpretar os resultados, sem repeti-los; o segundo, apresentar limitações e potenciais vieses. Evitar discussão genérica ou desconectada dos achados. Os resultados devem ser discutidos com base em literatura metodologicamente comparável, caracterizando os estudos citados (delineamento, local e tempo).

7. Ilustrações (tabelas e figuras): Devem seguir as normas editoriais da revista, com títulos autossuficientes, notas de rodapé padronizadas, categorização adequada das variáveis e formatação compatível com as exigências da publicação.

8. Recomendamos a revisão cuidadosa da redação científica, do estilo e da padronização terminológica, incluindo o uso correto de expressões como p-valor, covid-19.

9. É fundamental atender à política de dados abertos da RESS, indicando o repositório onde os dados analisados estão depositados, conforme as instruções editoriais da revista

Solicitamos que todos os comentários da revisão por pares sejam respondidos pontualmente por meio de uma carta resposta detalhada, explicando as modificações realizadas ou justificando, quando necessário, a manutenção do texto original. A nova versão do manuscrito será reconsiderada após essa reformulação e poderá passar por nova rodada de avaliação. Destacamos que esta decisão não garante a aceitação final do manuscrito.

Recommendation: Major revision

## Second round comments

Agradecemos a submissão do manuscrito RESS-2025-0405 R1. intitulado “Análise das características demográficas, microbianas e evolutivas de pacientes adultos com infecções relacionadas à assistência à saúde internados em unidade de terapia intensiva de Mato Grosso, 2018-2021.

Após a revisão do seu artigo, identificamos a necessidade de uma Revisão menor para que o manuscrito esteja de acordo com os padrões editoriais da revista e para viabilizar sua publicação.

1. Revisar o título e o objetivo do resumo e do corpo do texto para garantir alinhamento e clareza conceitual. Sugere-se que os autores explicitem o que entendem por “características evolutivas”. Caso não seja possível delimitar ou justificar adequadamente esse termo, recomenda-se sua retirada do título e do objetivo. Para mantê-lo, os autores devem ser capazes de: (1) definir claramente o conceito; (2) explicitar a metodologia adotada para sua análise; e (3) apresentar resultados coerentes com essa proposta.

Como alternativa, sugere-se reformular o título para:

“Análise das características demográficas, microbiológicas e desfechos clínicos de pacientes adultos com infecções relacionadas à assistência à saúde internados em unidade de terapia intensiva de Mato Grosso, 2018-2021.”

2. Revisar o resumo garantindo fidelidade e coerência com os dados apresentados no texto completo, especificamente: corrigir a descrição dos tipos de IRAS mais frequentes, distinguindo os períodos; esclarecer e quantificar corretamente os achados sociodemográficos e clínicos por biênio; epresentar de forma objetiva os percentuais de cura e óbito; quantificar as variações nos perfis microbianos e padrões de resistência.

3. Reduzir a extensão do resumo para até 250 palavras

4. A seção Discussão está muito extensa, repetitiva e fragmentada, com sobreposição de informações e ausência de parágrafo conclusivo.

Sugestão de estrutura para a Discussão:

• Parágrafo 1 - Interpretação crítica dos principais achados (síntese dos resultados mais relevantes, com hipóteses plausíveis e sem repetição numérica);

• Parágrafo 2 - Limitações e vieses (ex.: delineamento retrospectivo, uso de dados secundários, subnotificação e ausência de controle de confundidores);

• Parágrafo 3 - Comparação com estudos similares: tipos de IRAS e desfechos clínicos;

• Parágrafo 4 - Comparação com estudos sobre resistência antimicrobiana;

• Parágrafo 5 - Implicações práticas e para políticas públicas (vigilância epidemiológica e uso racional de antimicrobianos em contextos de crise sanitária);

• Parágrafo 6 - Conclusão da discussão (síntese final dos achados e importância prática).

5. Tabelas

• Alinhar à direita os títulos das colunas com dados numéricos, para manter consistência visual com os valores;

• Padronizar a notação como n (%), com “n” em minúscula;

• Substituir o hífen por “0” quando não houver casos;

• Separar claramente os blocos de variáveis (sexo, faixa etária, raça/cor da pele etc.) com recuo visual ou linha em branco;

• Substituir o termo genérico “Outros” por “Poliartrite” caso essa seja a única condição agrupada;

• Substituir o termo “desfecho evolutivo” por “desfecho clínico” no texto e nas tabelas, para maior precisão terminológica.

Solicitamos que todos os comentários sejam respondidos pontualmente por meio de uma carta resposta detalhada, explicando as modificações realizadas ou justificando, quando necessário, a manutenção do texto original. A nova versão do manuscrito será reconsiderada após essa reformulação e poderá passar por nova rodada de avaliação.

Recommendation: Minor revision

## Third round comments

Agradecemos a submissão do manuscrito RESS-2025-0405 R2 intitulado “Análise das características demográficas, microbiológicas e desfechos clínicos de pacientes adultos com infecções relacionadas à assistência à saúde internados em unidade de terapia intensiva de Mato Grosso, 2018-2021” à Revista Epidemiologia e Serviços de Saúde: revista do SUS (RESS).

Após a revisão, verificamos avanços relevantes em relação à versão anterior. Entretanto, alguns pontos ainda precisam ser ajustados para que o manuscrito esteja em conformidade com os padrões editoriais da RESS e viabilize sua publicação. Trata-se de ajustes de revisão menor, conforme detalhado a seguir.

-Título: recomendo que o ajustem para incluir, de forma explícita, o tema principal, o delineamento do estudo, o local e o(s) ano(s) da pesquisa. “Infecções relacionadas à assistência à saúde em unidade de terapia intensiva: estudo observacional retrospectivo em Mato Grosso, Brasil, 2018-2021”.

- Resumo deve ter até 250 palavras, no momento faltam elementos e o memso se encontra com 256 palavras

- Resumo (resultados): para garantir comparabilidade, ambos os períodos deveriam trazer os mesmos tipos de variáveis (sociodemográficas, clínicas e desfechos); precisam ser reorganizados para garantir clareza e comparabilidade. Solicita-se que apresentem, separadamente para os períodos pré-pandemia (2018-2019) e pandemia (2020-2021): (i) os tipos de IRAS mais frequentes, com percentuais; (ii) as características sociodemográficas de forma consistente (sexo, idade, raça/cor quando disponível); (iii) as principais comorbidades; e (iv) os desfechos clínicos, sempre informando conjuntamente percentuais de cura e óbito. Quanto ao perfil microbiano, descrevam de forma objetiva a taxa global de resistência a carbapenêmicos em cada período e, em seguida, resumam os principais microrganismos com seus percentuais, evitando frases longas e excesso de números, de modo a destacar apenas os achados centrais.

- Resumo (conclusão): reformulem para evidenciar, de forma sintética, a relevância clínica e epidemiológica das IRAS em UTI, ressaltando a implicação desses resultados para a assistência, vigilância e prevenção, sem repetir os dados numéricos já descritos nos resultados. Uma possibilidade é destacar a gravidade das infecções, a persistência de resistência microbiana e a necessidade de estratégias de controle mais efetivas no contexto hospitalar.

- Objetivo do estudo: reflita se o objetivo do estudo não é “Analisar as características demográficas, microbiológicas e desfechos clínicos de pacientes adultos com IRAS internados em unidade de terapia intensiva de Mato Grosso, entre 2018 e 2021, comparando os períodos pré-pandemia e pandemia de covid-19.” Se sim, o objetivo deve ser reformulado de maneira semelhante em resumo e introdução.

- Método: Reforçamos a necessidade de adequar o manuscrito ao checklist STROBE, que é a diretriz internacional para o relato de estudos observacionais. Devem ser apresentados, em subtópicos ou em parágrafos distintos, os seguintes itens: delineamento do estudo (especificando tratar-se de estudo observacional retrospectivo), contexto (local e período do estudo), participantes (critérios de inclusão e exclusão), variáveis analisadas (sociodemográficas, clínicas, microbiológicas e desfechos), fontes de dados e forma de mensuração, potenciais vieses e medidas adotadas para minimizá-los, tamanho do estudo (número total de participantes e justificativa, se aplicável) e métodos estatísticos utilizados (análises realizadas e softwares empregados).

- Delineamento do estudo: uniformizar a descrição do delineamento em todo o manuscrito para “estudo observacional retrospectivo”. O delineamento deve ser descrito de forma idêntica no título, resumo e corpo do texto.

- Resultados: Recomendamos: (i) manter nas tabelas os percentuais detalhados e, no texto, destacar apenas os achados centrais; (ii) padronizar a apresentação das variáveis sociodemográficas, clínicas e desfechos, de modo que os mesmos itens sejam descritos em ambos os períodos (pré-pandemia e pandemia), sempre informando conjuntamente os percentuais de cura e óbito; (iii) resumir a descrição dos microrganismos mais frequentes, evitando listas extensas no corpo do texto; e (iv) apresentar os resultados de resistência antimicrobiana de forma mais objetiva, por microrganismo e período, remetendo às tabelas para dados completos.

- Discussão: sugerimos alguns ajustes finais de aprimoramento: (i) reduzir a repetição de resultados já apresentados, priorizando a interpretação crítica; (ii) sintetizar as comparações com outros estudos, ressaltando as semelhanças e diferenças mais relevantes; (iii) organizar de forma mais objetiva a discussão sobre resistência antimicrobiana, evitando longas listas de microrganismos e percentuais; e (iv) tornar a conclusão mais concisa, limitando-se à contribuição central do estudo e às implicações práticas. Esses ajustes não configuram revisão maior, mas contribuirão para deixar a discussão mais clara, crítica e alinhada ao padrão da RESS.

- Tabela 1: padronizar a notação como n, com “n” em minúscula no título também.

Solicitamos que todos os comentários sejam respondidos pontualmente por meio de uma carta resposta detalhada, explicando as modificações realizadas ou justificando, quando necessário, a manutenção do texto original. A nova versão do manuscrito será reconsiderada após essa reformulação e poderá passar por nova rodada de avaliação.

Recommendation: Minor revision

## Fourth round comments

Agradecemos a submissão do manuscrito RESS-2025-0405 R3 intitulado “Infecções relacionadas à assistência à saúde em unidade de terapia intensiva: estudo observacional retrospectivo em Mato Grosso, Brasil, 2018-2021” à Revista Epidemiologia e Serviços de Saúde: revista do SUS (RESS).

Após a revisão, verificamos avanços relevantes em relação à versão anterior, com maior clareza na apresentação dos resultados e aprimoramentos na discussão. Entretanto, o objetivo do estudo ainda necessita de ajuste, conforme já havia sido apontado em parecer anterior. Trata-se de ajustes de revisão menor, conforme detalhado a seguir:

- Objetivo do estudo (resumo e texto completo): O(a) autor(a) não atendeu integralmente à recomendação de reformulação do objetivo. Observa-se que, tanto no resumo quanto no final da introdução, o objetivo foi mantido como “analisar as características demográficas, microbiológicas e desfechos clínicos de pacientes adultos com IRAS em UTI de Mato Grosso, entre 2018 e 2021”. Contudo, pelos métodos e resultados, nota-se que a análise foi conduzida de forma comparativa entre os períodos pré-pandemia (2018-2019) e pandemia de covid-19 (2020-2021).

Recomenda-se, portanto, reformular o objetivo no resumo e na introdução para que fique explícito e coerente com o delineamento do estudo e as análises realizadas.

Sugestão de redação:

“Analisar as características demográficas, microbiológicas e desfechos clínicos de pacientes adultos com infecções relacionadas à assistência à saúde (IRAS) internados em unidade de terapia intensiva de Mato Grosso, entre 2018 e 2021, comparando os períodos pré-pandemia e pandemia de covid-19.”

Recommendation: Minor revision

